# Supplementary material for: Structural basis of human ghrelin receptor signaling by ghrelin and the synthetic agonist ibutamoren
Source: Nat Commun. 2021 Nov 4;12:6410. doi: 10.1038/s41467-021-26735-5 (PMC8568970; doi:10.1038/s41467-021-26735-5)
Supplement: Supplementary file 1 — Supplementary Information [file 41467_2021_26735_MOESM1_ESM.pdf]

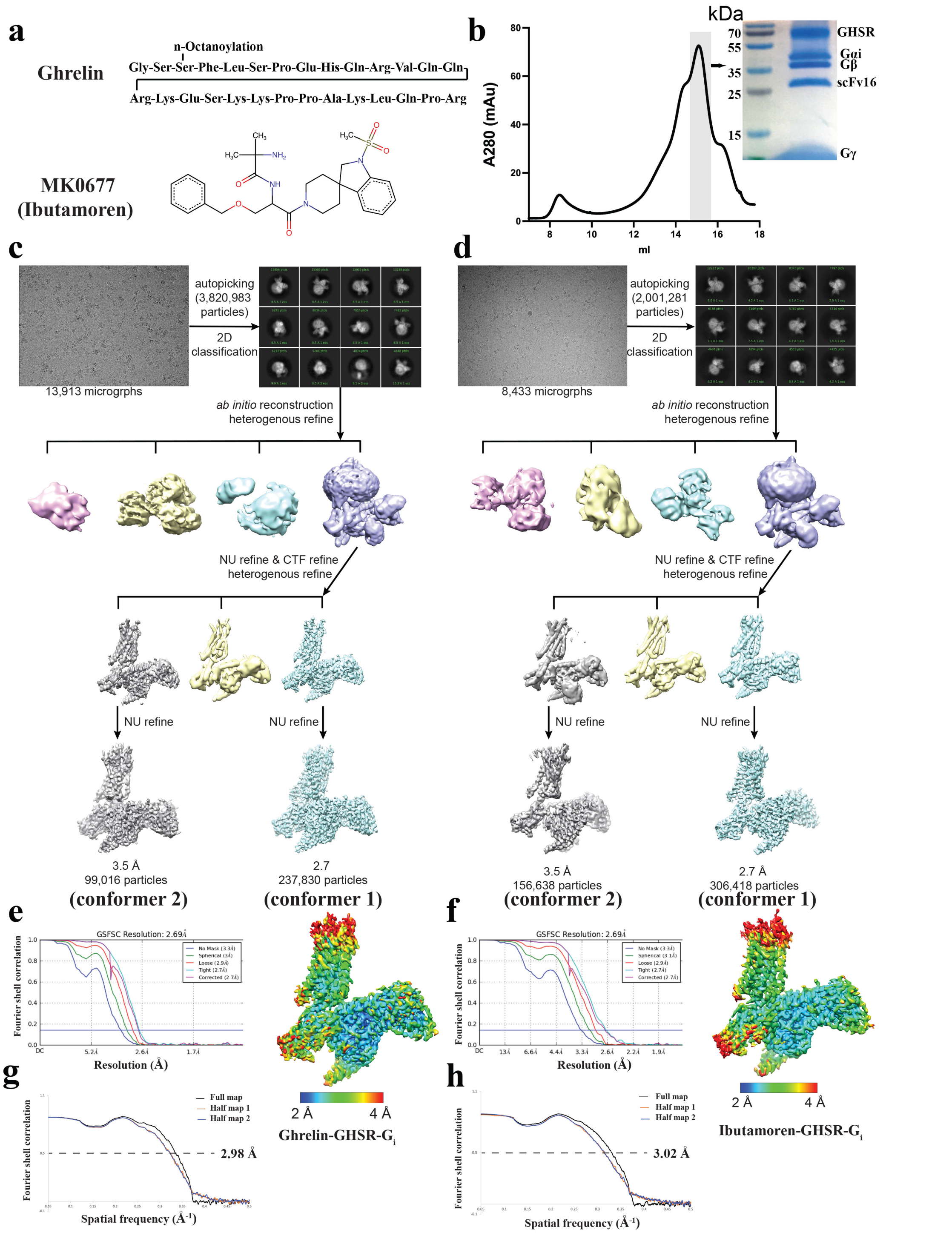

Supplementary Figure 1. Structure determination of the GHSR-G$_i$ complexes with ghrelin and ibutamoren. (a) Chemical structures of ghrelin and ibutamoren. **(b)** Size exclusion profile and SDS-PAGE analysis of the purified GHSR-G$_i$ complex with ghrelin. The original SDS-PAGE gel image can be found in the Source Data file. **(c)** and **(d)** Cryo-EM image processing workflow for the ghrelin-GHSR-G$_i$ complex (c) and the ibutamoren-GHSR-G$_i$ complex (d). **(e)**

and **(f)** Fourier shell correlation (FSC) curves with the estimated resolution according to the gold standard with a mask excluding detergent micelles and the local resolution estimates for the ghrelin-GHSR-G_i complex (e) and the ibutamoren -GHSR-G_i complex (f). **(g)** and **(h)** FSC curves of structural models of ghrelin-GHSR-G_i (g) and ibutamoren -GHSR-G_i (h) against the sharpened maps (Full map, black) and the half maps used (Half map 1, FSCwork, orange) and not used (Half map 2, FSCfree, blue) in the refinement. In both structures, the GHSR residues from P39 to F340 except for R244-R254 in the intracellular loop 3 (ICL3) and the whole G_i heterotrimer except for the α-helical domain (AHD) of $G_{\alpha i}$ were modeled. In the structure of GHSR with ibutamoren, the regions Y106-R107 and N188-D191 in the extracellular loops 1 and 2 (ECL1 and 2), respectively, are not modeled due to weak maps.

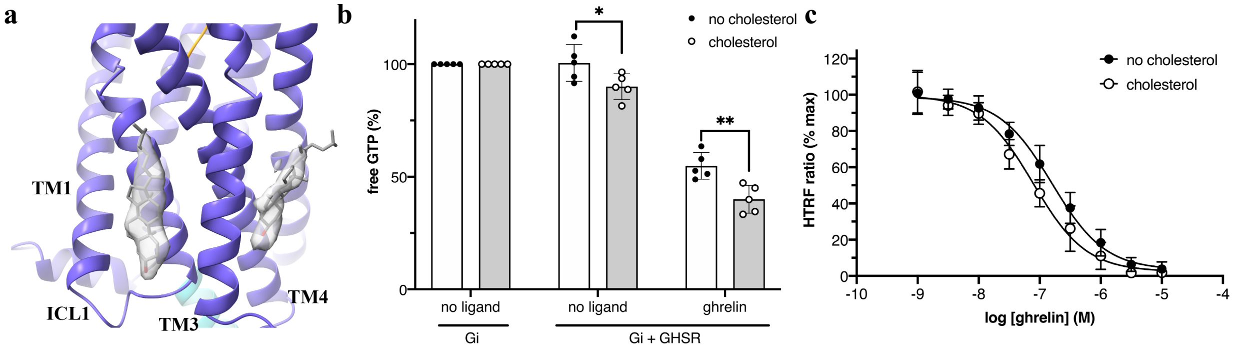

Supplementary Figure 2. Potential cholesterol binding sites and effects of cholesterol in GHSR signaling. (a) Cryo-EM density maps of two cholesterol molecules modeled in the structure of ghrelin-GHSR-G_i.GHSR is colored in slate. Cholesterol molecules are shown as grey sticks. **(b)** GTP turnover for G_i catalyzed by wild type GHSR in nanodiscs containing 10% cholesterol (molar ratio) or not, in the absence or presence of 10 µM ghrelin. The signal was normalized to that obtained for the G protein in the absence of receptor (Gi panels), and data is mean ± SD of five experiments. Statistical values were obtained by means of two-sided Student's t test (*0.01<p<0.05, **0.001<p<0.01; p=0.0493 and 0.0047 for the apo and ghrelin-loaded receptor, respectively). Cholesterol showed subtle but significant positive effects in enhancing GHSR-induced G_i activation both with and without ghrelin. **(c)** HTRF-monitored competition experiments between dy647-JMV2959 and ghrelin for binding wild type GHSR in the absence or presence of 10% cholesterol (molar ratio) into the lipid discs. Each data point represents mean ± SD of three experiments.

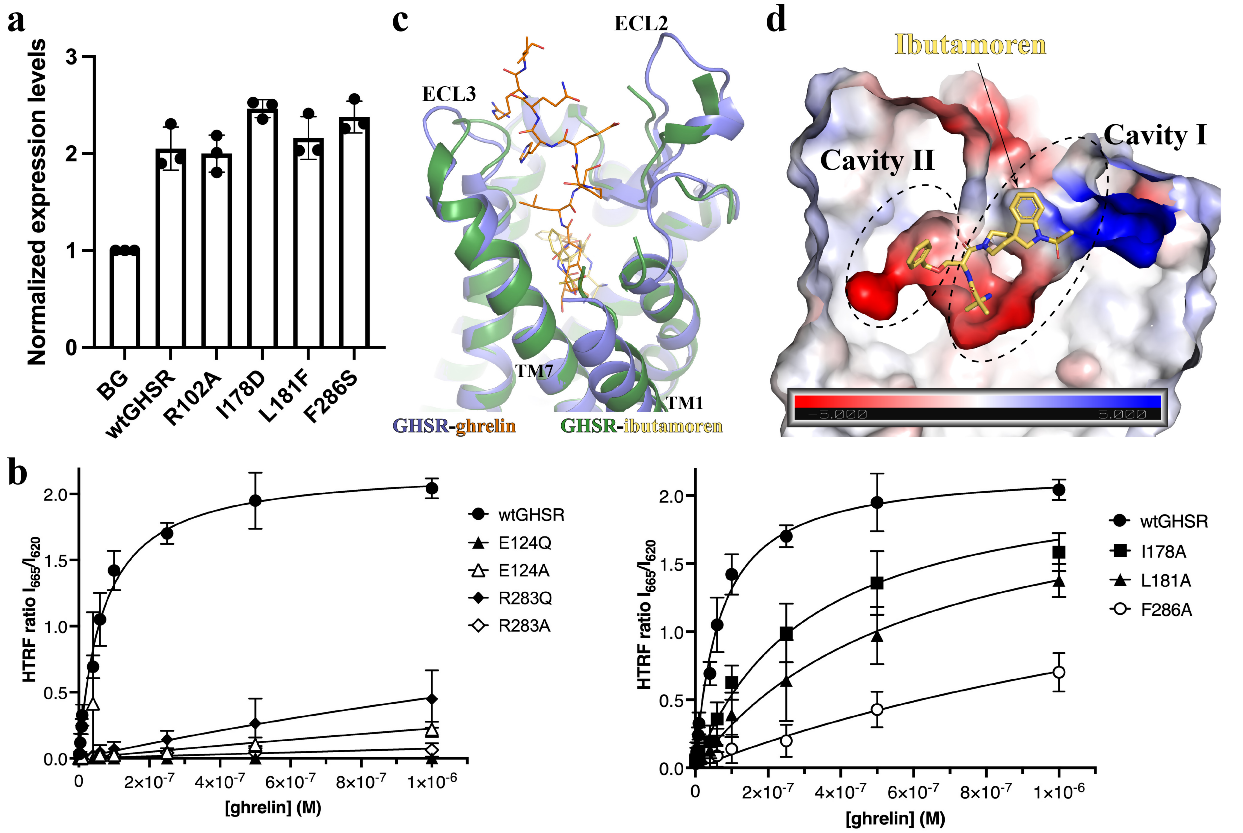

Supplementary Figure 3. Ghrelin and ibutamoren binding pockets and expression of GHSR mutants. (a) Surface expression levels of GHSR mutants tested in **Figure 2**. For each GHSR mutant, the expression level was determined by measuring the fluorescence intensity of transfected HEK-293 cells stained with a fluorescent anti-FLAG antibody that could bind to the N-terminal FLAG peptide of each GHSR construct. BG means background, representing results from non-transfected HEK-293 cells. Each column represents results shown as mean ± s.d. from 3 wells of cells transiently transfected with each GHSR mutant. **(b)** HTRF-monitored binding of dy647-labeled ghrelin to the wild type GHSR (wtGHSR) and mutants assembled into POPC:POPG lipid discs. The binding data are presented as variations in the HTRF ratio as a function of labeled ghrelin concentration. Each data point represents mean ± SD of three experiments. **(c)** Alignment of the extracellular regions of GHSR bound to ghrelin (orange) and ibutamoren (yellow). The receptors are colored in slate and green, respectively. ECL2 and ECL3 show notable conformational differences. **(d)** Charge distribution of the ibutamoren-binding pocket. Cavity I and II are circled.

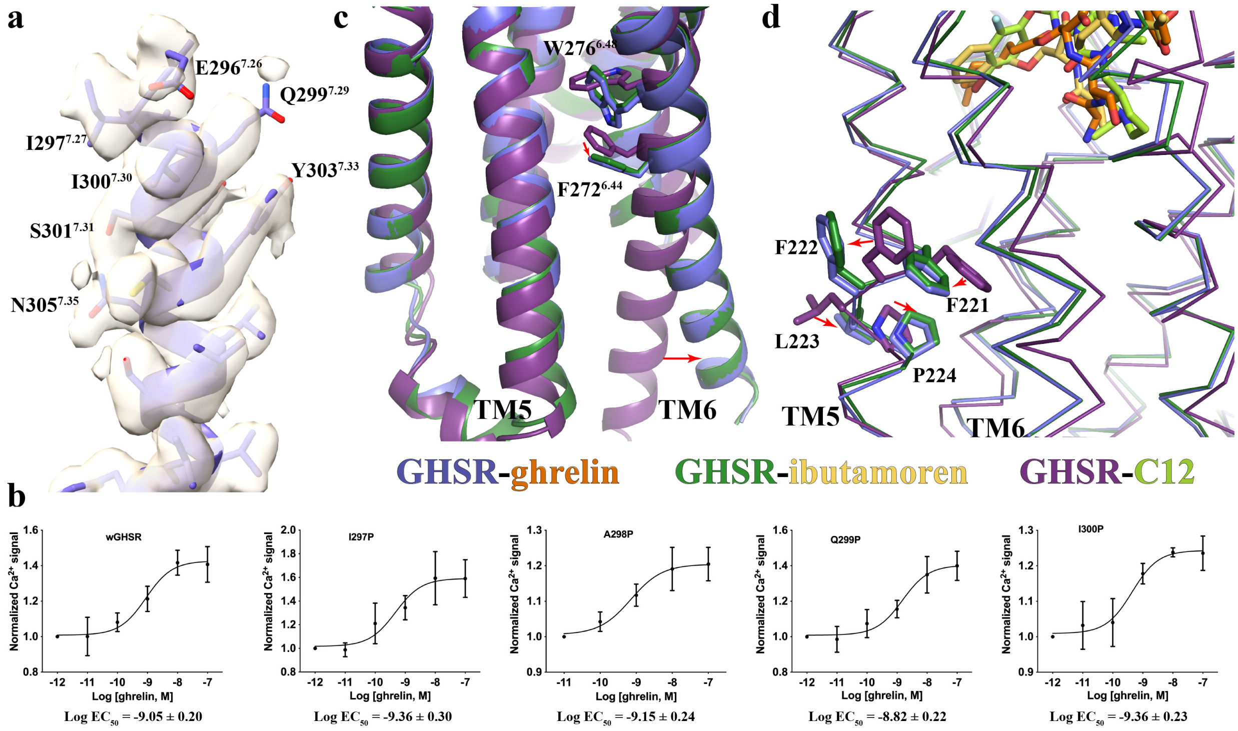

Supplementary Figure 4. Conformational changes of residues in the core region of active GHSR bound to two agonists compared to them in the inactive GHSR bound to C12. (a) Cryo-EM density map of the N-terminal region of TM7 in the structure of ghrelin-bound GHSR. **(b)** Dose-dependent action of ghrelin on the wild type GHSR (wtGHSR) and mutants I297P, A298P, Q299P, and I300P. Each data point represents Mean ± S. D. from 3 independent assays. The $LogEC_{50}$ values represent best-fit values and standard errors. The four mutations didn't significantly affect the EC_{50}s of ghrelin in inducing GHSR signaling. **(c)** Conformational changes of the highly conserved transmission switch $F272^{6.44}$ and $W276^{6.48}$ and accompanied conformational change of TM6. Numerous studies suggested that conformational changes of these two residues link extracellular agonist-binding events to the activation of GPCRs at cytoplasmic regions for G protein-coupling. **(d)** Conformational changes of residues in the core region of TM5. Red arrows indicate changes of residues from the inactive to the active states. Two active GHSR and the inactive GHSR structures are colored in blue, green, and purple, respectively. Ghrelin, ibutamoren and C12 are shown as orange, yellow and lemon sticks, respectively.

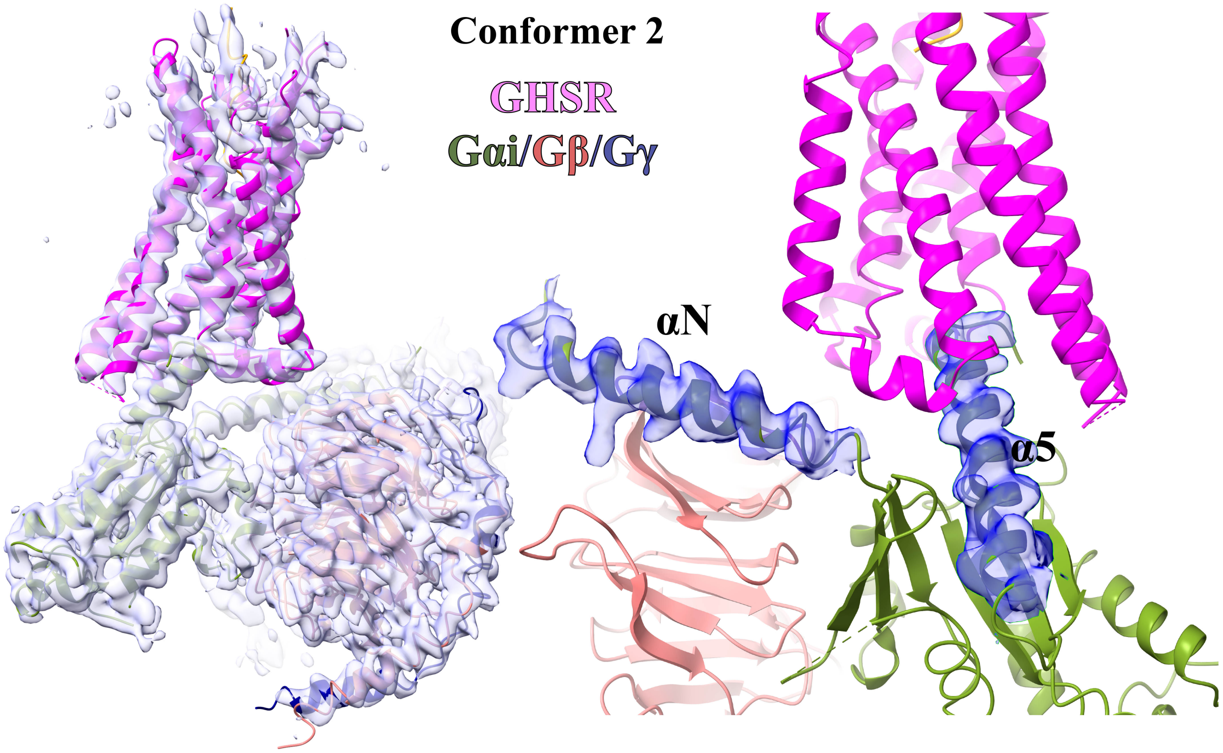

Supplementary Figure 5. Cryo-EM density maps of the ghrelin-GHSR-G_i complex in Conformer 2. The density of the entire ghrelin-GHSR-G_i complex is shown in the left panel. The density of α5 and αN of $G_{\alpha i}$ is shown in the right panel. GHSR, $G_{\alpha i}$, G_{β} and G_{γ} are colored in violet, forest, ruby, and deep blue, respectively.

Supplementary Table 1. Cryo-EM data collection, structure refinement and statistics

	**Ghrelin-GHSR-G_i-scFV16** (EMD-24267, PDB 7NA7)	**Ibutamoren-GHSR-G_i-scFV16** (EMD-24268, PDB 7NA8)
**Data acquisition**		
Microscope/Camera	Titan Krios/Gatan K3 Camera	
Voltage (kV)	300	
Defocus range (μM)	0.8-1.6	0.8-1.6
Pixel size (Å)	0.649	0.826
Total electron dose (e^-/Å^2)	82.3	81.0
Exposure time (s)	2.1	3
**Reconstruction**		
Particle number	280,046	283,300
Resolution (masked, Å)	2.7	2.7
**RMS deviation**		
Bond length (Å)	0.006	0.006
Bond angle (°)	1.042	0.864
**Ramachandran plot**		
Favored (%)	98.39	96.91
Allowed (%)	1.61	3.09
Outliers (%)	0	0
**MolProbity**		
Clash score	6.71	7.77
Rotamer outliers (%)	0.61	0.31

Supplementary Table 2. Primers used in this study.

Primers for cloning of GHSR into pFastBac. 5'-3'.	
GHSRForwardSalI	TTCCAATCAGTCGACTGGAACGCGACGCCCAGC
GHSRReverseNotI	ACTATTCTAATGCGGCCGCTGTATTAATACTAGATTCTGTCCAGGCCCG
Primers for cloning of GHSR into pcDNA3.1+. 5'-3'.	
GHSRBNEcoRI	ATTACTGAATTCATGGACTCGAAGGGCTCGTC
GHSRBNXhoI	TGATTGGAACTCGAGTCAGTGGTGATGATG
Primers for generating GHSR mutations. 5'-3'.	
R102AFor	pho-GCGCTCTGGCAGTACCGGCCCTGG
R102ARev	pho-AACGAGGTCCAGGGGCATGCA
I178DFor	pho-GACTTCGTGCTAGTCGGGGTGGAG
I178DRev	pho-GGGCCCGGCGCTGCAGAAGGCCAC
L181FFor	GGCCCATCTTCGTGTTTGTCGGGGTGGAGCA
L181FRev	TGCTCCACCCCGACAAACACGAAGATGGGCC
F286SFor	CCCTTCCACGTAGGGCGATATTTATCTTCCAAATCCTTTG
F286SRev	CAAAGGATTTGGAAGATAAATATCGCCCTACGTGGAAGGG
I297PFor	pho-GCTCAGATCAGCCAGTACTGCAA
I297PRev	pho-AGGCTCCAAGGAGCCAGGCTC
A298PFor	pho-CAGATCAGCCAGTACTGCAA
A298PRev	pho-CGGAGCAATCTCCAAGGAGCC
Q299PFor	pho-ATCAGCCAGTACTGCAACCTC
Q299PRev	pho-CGGAGCAATCTCCAAGGAGCC
I300PFor	pho-GGGCTGAGCAATCTCCAAGGAGC
I300PRev	pho-AGCCAGTACTGCAACCTCGT
