# Supplementary material for: Structural basis of human ghrelin receptor signaling by ghrelin and the synthetic agonist ibutamoren
Source: Nat Commun. 2021 Nov 4;12:6410. doi: 10.1038/s41467-021-26735-5 (PMC8568970; doi:10.1038/s41467-021-26735-5)
Supplement: Supplementary file 3 — Reporting summary [file 41467_2021_26735_MOESM3_ESM.pdf]

nature portfolio

Corresponding author(s): Ji Sun, Cheng Zhang

Last updated by author(s): Sep 9, 2021

Reporting Summary

Nature Portfolio wishes to improve the reproducibility of the work that we publish. This form provides structure for consistency and transparency in reporting. For further information on Nature Portfolio policies, see our Editorial Policies and the Editorial Policy Checklist.

Statistics

For all statistical analyses, confirm that the following items are present in the figure legend, table legend, main text, or Methods section.

n/a	Confirmed	
☐	☒	The exact sample size (n) for each experimental group/condition, given as a discrete number and unit of measurement
☐	☒	A statement on whether measurements were taken from distinct samples or whether the same sample was measured repeatedly
☐	☒	The statistical test(s) used AND whether they are one- or two-sided<br>*Only common tests should be described solely by name; describe more complex techniques in the Methods section.*
☒	☐	A description of all covariates tested
☒	☐	A description of any assumptions or corrections, such as tests of normality and adjustment for multiple comparisons
☐	☒	A full description of the statistical parameters including central tendency (e.g. means) or other basic estimates (e.g. regression coefficient) AND variation (e.g. standard deviation) or associated estimates of uncertainty (e.g. confidence intervals)
☐	☒	For null hypothesis testing, the test statistic (e.g. F, t, r) with confidence intervals, effect sizes, degrees of freedom and P value noted<br>*Give P values as exact values whenever suitable.*
☒	☐	For Bayesian analysis, information on the choice of priors and Markov chain Monte Carlo settings
☒	☐	For hierarchical and complex designs, identification of the appropriate level for tests and full reporting of outcomes
☒	☐	Estimates of effect sizes (e.g. Cohen's d, Pearson's r), indicating how they were calculated

Our web collection on statistics for biologists contains articles on many of the points above.

Software and code

Policy information about availability of computer code

Data collection	SerialEM
Data analysis	cryoSPARC, MotionCor2, GCTF, Chimera, Coot 0.9, Phenix, Molprobity, GraphPad Prism 8.

For manuscripts utilizing custom algorithms or software that are central to the research but not yet described in published literature, software must be made available to editors and reviewers. We strongly encourage code deposition in a community repository (e.g. GitHub). See the Nature Portfolio guidelines for submitting code & software for further information.

Data

Policy information about availability of data

All manuscripts must include a data availability statement. This statement should provide the following information, where applicable:

- Accession codes, unique identifiers, or web links for publicly available datasets
- A description of any restrictions on data availability
- For clinical datasets or third party data, please ensure that the statement adheres to our policy

The 3D cryo-EM density maps of the ghrelin-GHSR-Gi complex and the ibutamoren-GHSR-Gi complex generated in this study have been deposited in the Electron Microscopy Data Bank database under accession codes EMD-24267 and EMD-24268, respectively. The atomic coordinates for the atomic models of the ghrelin-GHSR-Gi and ibutamoren-GHSR-Gi complexes generated in this study have been deposited in the Protein Data Bank database under accession codes 7NA7 and 7NA8, respectively. The raw data for the main Figure 2c and f and Supplementary Figure 2b and c, 3b, and 4b generated in this study are provided in the Source Data file. The structural models of GHSR with the antagonist C12 and the neurotensin-NTSR-Gi complex used in this study are available in the Protein Data Bank database under accession codes 6KO5 (http://doi.org/10.2210/pdb6ko5/pdb) and 6OS9 (http://doi.org/10.2210/pdb6OS9/pdb). Source data are provided with this paper.

Field-specific reporting

Please select the one below that is the best fit for your research. If you are not sure, read the appropriate sections before making your selection.

☒ Life sciences ☐ Behavioural & social sciences ☐ Ecological, evolutionary & environmental sciences

For a reference copy of the document with all sections, see nature.com/documents/nr-reporting-summary-flat.pdf

Life sciences study design

All studies must disclose on these points even when the disclosure is negative.

Sample size	For all signaling and binding assays, we used data from 3-5 experiments because three biological replicates are the minimum for inferential analysis.
Data exclusions	None.
Replication	For all signaling and binding assays, we used data from 3-5 repeated experiments . Not all attempts at replication were successful because of mistakes in sample preparation.
Randomization	For each dataset (curves) in our binding and signaling assays, cells were uniformly seeded in plates before treatment to ensure comparable backgrounds.
Blinding	The investigators were aware of how cells were treated before collecting data. Data were collected from seeded cells in multi-well plates in an automated manner. No biased results were introduced by the investigators.

Reporting for specific materials, systems and methods

We require information from authors about some types of materials, experimental systems and methods used in many studies. Here, indicate whether each material, system or method listed is relevant to your study. If you are not sure if a list item applies to your research, read the appropriate section before selecting a response.

Materials & experimental systems

n/a	Involved in the study	
☐	☒	Antibodies
☐	☒	Eukaryotic cell lines
☒	☐	Palaeontology and archaeology
☒	☐	Animals and other organisms
☒	☐	Human research participants
☒	☐	Clinical data
☒	☐	Dual use research of concern

Methods

n/a	Involved in the study	
☒	☐	ChIP-seq
☒	☐	Flow cytometry
☒	☐	MRI-based neuroimaging

Antibodies

Antibodies used	Anti-FLAG M1 antibody
Validation	Western blot using FLAG-tagged proteins. Purification of FLAG-tagged receptors by M1 antibody-conjugated resin.

Eukaryotic cell lines

Policy information about cell lines

Cell line source(s)	Expression Systems
Authentication	None of the cell lines used were authenticated.
Mycoplasma contamination	The cell lines were not tested for mycoplasma contamination
Commonly misidentified lines (See ICLAC register)	None found in the ICLAC database.
